# Val103Ile polymorphism of the melanocortin-4 receptor gene (*MC4R*) in cancer cachexia

**DOI:** 10.1186/1471-2407-8-85

**Published:** 2008-03-31

**Authors:** Susanne Knoll, Sabiene Zimmer, Anke Hinney, André Scherag, Andreas Neubauer, Johannes Hebebrand

**Affiliations:** 1 Department of Child and Adolescent Psychiatry, University of Duisburg-Essen, Germany; 2 Department of Hematology/Oncology/Immunology, University of Marburg, Germany; 3 Zentrum for clinical studies food (ZKSE) c/o Institute for Medical Informatics, Biometry and Epidemiology, University Duisburg-Essen, Essen, Germany

## Abstract

**Background:**

At present pathogenic mechanisms of cancer cachexia are poorly understood. Previous evidence in animal models implicates the melanocortin-4 receptor gene (*MC4R*) in the development of cancer cachexia. In humans, *MC4R *mutations that lead to an impaired receptor function are associated with obesity; in contrast, the most frequent polymorphism (Val103Ile, rs2229616; heterozygote frequency approximately 2%) was shown to be negatively associated with obesity. We tested if cancer patients that are homo-/heterozygous for the Val103Ile polymorphism are more likely to develop cachexia and/or a loss of appetite than non-carriers of the 103Ile-allele.

**Methods:**

BMI (body mass index in kg/m^2^) of 509 patients (295 males) with malignant neoplasms was determined; additionally patients were asked about premorbid/pretherapeutical changes of appetite and weight loss. Cachexia was defined as a weight loss of at least 5% prior to initiation of therapy; to fulfil this criterion this weight loss had to occur independently of other plausible reasons; in single cases weight loss was the initial reason for seeing a physician. The average age in years (± SD) was 59.0 ± 14.5 (males: 58.8 ± 14.0, females 59.2 ± 14.0). Blood samples were taken for genotyping of the Val103Ile by PCR- RFLP.

**Results:**

Most of the patients suffered from lymphoma, leukaemia and gastrointestinal tumours. 107 of the patients (21%) fulfilled our criteria for cancer cachexia. We did not detect association between the Val103Ile polymorphism and cancer cachexia. However, if we exploratively excluded the patients with early leucaemic stages, we detected a trend towards the opposite effect (p < 0.05); heterozygotes for the 103Ile-allele developed cancer cachexia less frequently in comparison to the rest of the study group. Changes of appetite were not associated with the 103Ile-allele carrier status (p > 0.39).

**Conclusion:**

Heterozygotes for the 103Ile-allele are not more prone to develop cancer cachexia than patients without this allele; possibly, Ile103 carriers might be more resistant to cancer cachexia in patients with solid tumors. Further studies of the melanocortinergic system in cachexia of patients with solid tumors are warranted.

## Background

The cachexia syndrome represents a complex metabolic state accompanied by muscle wasting and a loss of body fat, hence quality of life is deteriorated and the prognosis of the patients who suffer from it is reduced [1,2]. Reports mostly vary in the amount of weight loss (5–10%) and the time span (6–12 months) in which the weight loss occurs [3-7]. Up to one-half of untreated cancer patients is expected to lose weight while about one third is expected to lose more than 5%; about 20% of cancer deaths may be due to cachexia [3]. Anorexia, defined as the loss of appetite and early satiety, is present in up to one-half of newly diagnosed cancer patients [8]. In addition, a number of non-specific factors associated with cancer (e.g. changes in taste and smell, vomiting, pain) may contribute to limited food intake. However, loss of appetite is not obligatory for the development of cachexia [4].

Although a formal classification for cancer cachexia does not exist, it can be presumed that a variable interaction of tumour products, neuroendocrine changes, and host inflammatory molecules leads to this observable wasting [5]. At present the mechanisms in the pathogenesis of cancer cachexia are still poorly understood. Here, the potential role of the melanocortin-4 receptor gene (*MC4R*) was investigated.

The MC4R is part of the leptinergic-melanocortinergic pathway that controls energy homeostasis. Stimulation of MC4R leads to higher energy expenditure, loss of appetite and weight loss [9]. A series of experiments demonstrated that cancer cachexia is ameliorated by central MC4R blockade in MC4R knock out mice or by peripheral administration of an antagonist (e.g. agouti-related protein – AGRP) in rats, mice and sheep [9-13]. Orally bioavailable potent antagonists of the human MC4R (e.g. synthesized compounds of Pyrrolidinones) demonstrated *in vivo *efficacy in protecting against cachectic symptoms in animal models of tumour-induced wasting and may be a suitable approach for the treatment of cachexia [14,15].

In humans, mutations that reduce the function of the MC4R result in severe obesity [16-18]. The Val103Ile polymorphism is the most common *MC4R *variant, with allele frequencies > 1% in almost all studied populations [17,19-22]. This polymorphism was repeatedly shown to be negatively associated with above average weight and obesity in humans [21-23]. Recently, the initial finding (Geller et al., 2004) was replicated in an independent sample [24] comprising 5.603 individuals. Young et al. (2007) confirmed these findings in a meta-analysis comprising 29,563 individuals: individuals harbouring the Ile allele had an 18% lower risk of obesity compared with non-carriers [23]. The Ile103 variant is assumed to entail a higher receptor activity [24,25].

Our primary objective was to evaluate the role of the *MC4R *Val103Ile polymorphism in the development of cachexia in patients with cancer. We hypothesized that cancer patients with the 103Ile-allele of the *MC4R *are more likely to develop cancer cachexia than patients without the respective allele. Furthermore we analyzed if loss of appetite independent of therapy and thus disease-related, is associated with the 103Ile-allele.

## Methods

### Subjects

509 consecutive patients from the outpatient unit of the Department of Hematology, Oncology and Immunology of the Philipps-University of Marburg were recruited from October 2003 until November 2004. Written informed consent was given by all participants. This study was approved by the ethics committee of the Philipps-University of Marburg.

### Questionnaire

Currently, there is no valid questionnaire to evaluate the status of cachexia in cancer patients. Thus, a new semi-structured interview was developed. Attention was paid to the weight-changes that had occurred prior to initiation of the cancer therapy. As a marker for cachexia we referred to pretherapeutical weight loss if it occurred a) 1–12 months prior to initiation of therapy and b) was not due to mechanical obstruction of the oro-gastrointestinal tract, c) could not readily be explained by a psychiatric disorder (such as depression) or d) an intentional diet. Besides data like date of birth, sex, type of cancer, stage of disease and therapy the interview included questions about weight, weight changes and appetite. Current height and weight were measured. Often self-reports are the only source of information about body weight in the past. Several studies have examined factors affecting the validity of self- reported past body weight [26-28]. The recalls partly were influenced by age, sex, the passage of time, the accuracy of current weight reports, weight gain and loss, and weight variability [26,28]. Nevertheless, past body weights were recalled with good accuracy in all of these studies.

### Definition of cachexia in cancer patients

As definitions of cancer cachexia vary and as no generally accepted standard exists [e.g. [3-7]], the patients were categorized in five different groups according to cachexia severity type. Type 1–3 indicate cachexia, whereas type 4 and 5 indicate a non-cachectic situation:

Type 1: *definitely cachectic*: weight loss of at least 10% of pretherapeutical weight that cannot be explained by other plausible reasons or which was the initial reason for seeing a physician. Weight loss as a consequence of therapy or oro-gastrointestinal obstruction (stenosis, etc.) could definitely be ruled out. 44 of the 509 patients (8.6%) were included in this type.

Type 2: *cachexia very probable*: weight loss of at least 10% of pretherapeutical weight that cannot be explained by other plausible reasons or which was the initial reason for seeing a physician. In contrast to type 1 we were not able to completely exclude that some weight loss was due to therapy (chemotherapy or radiation) or tumour related mechanical reasons. 20 patients (3.9%) were included in type 2.

Type 3: *cachexia probable*: weight loss of at least 5% but less than 10% of pretherapeutical weight that can not be explained by other plausible reasons or which was the initial reason for seeing a physician. In contrast to type 1 we were not able to completely exclude that some weight loss was due to therapy (chemotherapy or radiation) or tumour related mechanical reasons. 43 patients (8.4) were included in type 3.

Type 4: *cachexia unlikely*: a) weight loss of less than 5% or b) weight loss in excess of 5% of pretherapeutical weight that is most likely due to therapy (chemotherapy, radiation, etc.) or a mechanical reason (weight loss occurred after initiation of therapy; patient reported that obstruction led to reduction in food intake). 221 patients (43.4%) were included in type 4.

Type 5: *cachexia ruled out*: a) no weight loss or b) weight gain. 181 patients (35.6%) were included in type 5.

### Classification of the tumor progression

The different tumour types were divided into different classifications and stages that are related to prognostic values and are commonly used to define therapy options (Table 1). In detail: for patients with colorectal or other gastrointestinal cancers, breast and lung cancer we have used the Union international contre le cancer classification system [29]. We have used the World Health Organization (WHO) classification for subtypes of lymphomas [30], the stages were defined according to the Ann Arbor classification system [31], except for chronic lymphatic leukaemia (CLL) which were classified according to the classification of Binet [32]; and multiple myeloma which were classified according to Durie and Salmon [33]. We divided patients with all different above mentioned tumour types (except for CLL and multiple myeloma) into two groups: stage I/II (limited/intermediate diseases) and stage III/IV (advanced diseases). All other analyzed tumour types (prostate cancer, Primitive Neuroectodermal Tumour (PNET), Carcinoma of unknown primary (CUP) and other (rare) tumour types) were each to rare to analyze them according to their stages.

**Table 1:** Different diagnoses and stages within the study group and the proportion of patients with cancer cachexia (types 1–3; see *Methods*)

**Type of cancer**	**N patients**	**Of these cachectic**	**I/II^1,2 ^or related stages**	**Of these cachectic**	**III/IV^1,2 ^or related stages**	**Of these cachechtic**	**No data about stage**	**n female**	**Of these cachectic**	**n male**	**Of these cachectic**
**Lymphoma^1^**	202 *(40)*	**35 *(17.3)***	57+	**9 *(15.8)***	61+	**22 *(36.1)***	16+	84	**18 *(21.4)***	118	**17 *(14.4)***
**Colorectal^2^**	84 *(16)*	**23 *(27.4)***	9	**3 *(33.3)***	71	**19 *(26.8)***	4	33	**7 *(21.2)***	51	**16 *(31.4)***
**Leukaemia^3^**	58 *(11)*	**7 *(12.1)***	*		*		*	22	**4 *(18.2)***	36	**3 *(8.3)***
**Other^2 ^Gastrointestinal**	39 *(8)*	**15 *(38.5)***	4	**1 *(25)***	21	**8 *(38.1)***	14	8	**3 *(37.5)***	31	**12 *(38.7)***
**Lung^2^**	36 *(7)*	**9 *(25)***	6	**1 *(16.7)***	27	**8 *(29.6)***	3	9	**3 *(33.3)***	27	**6 *(22.2)***
**Breast^2^**	32 *(6)*	**4 *(12.5)***	17	**2 *(11.8)***	9	**2 *(22.2)***	6	32	**4 *(12.5)***	0	
**Leukaemia-early stages^3,4^**	20 *(4)*	**5 *(25)***	*		*		*	13	**2 *(15.4)***	7	**3 *(42.9)***
Prostate*	4 *(1)*	**1 *(25)***	*		*		*			4	**1 *(25)***
Others*	34 *(7)*	**8 *(23.5)***	*		*		*	13	**5 *(38.5)***	21	**3 (14.3)**
**Total**	509	**107 *(21)***	*	*	*	*	*	**214**	**46 *(21.5)***	**295**	**61 *(20.7)***

Numbers in parentheses denote the particular percentage.

^1^Lymphoma subgroups according to WHO (2001) and Ann Arbor classifications.

+The 24 patients with CLL (classification of Binet) and the 44 patients with multiple myeloma (classification of Durie and Salmon) were excluded.

^2^UICC classification was used for colorectal and other gastrointestinal tumors, breast and lung cancer.

^3^The different classifications for leucaemia and the leukaemia-early stages could not be used for these groups.

^4^Patients with myelodysplastic syndromes (MDS) or myeloproliferative syndromes (MPD).

*The classifications for these tumour types could not be used for subgrouping.

### Molecular genetic analysis

Blood samples were taken from all patients. DNA extraction and genotyping of the Val103Ile polymorphism was performed by specific PCR-RFLP as described previously [34].

### Statistical Analyses

To test for association of cachexia status (types 1–3 vs. types 4,5) with the Val103Ile *MC4R *polymorphism, the genotype frequencies in the respective groups were compared by Fisher's exact test. As only one hypothesis is tested α was set to 0.05 (two-sided). For exploratory purposes, post-hoc and sub-group analyses were conducted. The genotype distribution in the total sample was tested for deviations from Hardy Weinberg equilibrium (p = 1, exact test). Assuming a minor allele frequency of 0.05, a frequency of 0.2 for the type 1–3 cachexia status, and α = 0.05 (two-sided, Fisher's exact test), the total sample sizes of 509 individuals was estimated to yield a power of ≈0.80 to detect an odds ratio of ≈3.0. Hence the study is well powered to detect strong associations between carrier status and cachexia status.

## Results

The different malignant diseases that were diagnosed in our study group and the percentages of cachexia are shown in Table 1. Our results are consistent with previous reports that weight loss is more common in patients with lung and gastrointestinal cancer and less common in patients with breast cancer [3,35]. Patients with malignant neoplasms according to ICD 10, including 14 patients with myelodysplastic syndromes (MDS) and six patients with myeloproliferative syndromes (MPD: polycythemia vera, essential thrombocythemia, osteomyelofibrosis) were included; these preleukaemic stages can transform into acute myeloic leukaemia. Most of the patients suffered from lymphoma, leukaemia and gastrointestinal tumours.

The average age in years (± SD) was 59.0 ± 14.5 (males: 58.8 ± 14.0, females 59.2 ± 14.0). Average BMI of all 509 patients one year before diagnosis of cancer was 26.0 ± 3.4 kg/m^2^, at the time of diagnosis (prior to initiation of therapy) 25.5 ± 3.7 kg/m^2 ^and at ascertainment 25.3 ± 3.9 kg/m^2^. 107 patients (21%) developed cancer cachexia (types 1–3, see *Methods*).

### MC4R Val103Ile polymorphism

Among the total of 509 patients 25 (4.9%; average age 57.1 ± 16.6 years; 17 males) were heterozygous for the Val103Ile polymorphism. Of these 2 developed cancer cachexia (these two had non-neoplastic early leukaemic stages). Of the remaining 484 patients with the wild type genotype, 105 (21.7%) developed cancer cachexia (Table 2). In contrast to our primary hypothesis, there was no evidence for an association of the Ile103 allele with cancer cachexia (Fisher's exact test, two sided, p-value = 0.13). An exploratory post-hoc exclusion of the 20 patients with early leukaemic stages (Table 3), that can strictly be viewed as non-neoplasms even revealed a trend towards a negative association between the 103Ile polymorphism and cancer cachexia (Fisher's exact test, two sided, p-value = 0.0068).

**Table 2:** Patients heterozygous for the *MC4R *Val103Ile polymorphism, grouped according to tumour type; presence or absence of cachexia is shown

**Type of cancer**	**Heterozygotes for the 103Ile-allele**	**cachectic**	**I/II^1,2 ^or related stages**	**III/IV^1,2 ^or related stages**	**No data about stage**	**n female**	**n male**
**Lymphoma**	9	0	5	4	0	3	6
**Colorectal**	7	0	1	6	0	2	5
**Leukaemia**	2	0	*	*	*	0	2
**Other gastrointestinal**	1	0	0	1	0	0	1
**Lung**	1	0	1	0	0	0	1
**Breast**	2	0	2	0	0	2	0
**Leukaemia-early stages**	2	100%	*	*	*	1	1
**Prostate**	0	0	*	*	*	0	0
**Others**	1 (PNET^5^)	0	*	*	*	0	1

**Total**	**25**	**8%**	*	*	*	**8**	**17**

^1^Lymphoma subgroups according to WHO (2001) and Ann Arbor classifications.

+The 24 patients with CLL (classification of Binet) and the 44 patients with multiple myeloma (classification of Durie and Salmon) were excluded.

^2^UICC classification was used for colorectal and other gastrointestinal tumors, breast and lung cancer.

^3^The different classifications for leucaemia and the leukaemia-early stages could not be used for these groups.

^4^Patients with myelodysplastic syndromes (MDS) or myeloproliferative syndromes (MPD).

^5^PNET: Primitive Neuroectodermal Tumour

*The classifications for these different tumour types could not be used for subgrouping.

**Table 3:** Heterozygotes for the *MC4R *103Ile-allele and cachexia status (as defined in *Methods*)

**Patients with**	**Cachexia** **N = 107 *(21)*** **[102]**	**No cachexia** **N = 402 *(79)*** **[387]**	**Total** ** N = 509** **[489]**
**Heterozygotes for the 103Ile-allele**	2 [0]	23 [23]	25 [23]
**No Heterozygotes for the 103Ile-allele**	105 [102]	379 [364]	484 [466]

Numbers in square brackets denote the number of patients without early leucaemic preliminary stages.

*Numbers in parentheses denote the particular percentage*.

Fisher's exact test, two sided, p-value = 0.13 [0.0068]

### The role of changes in appetite

232 (45.6%) of the 509 patients reported a general loss of appetite (for details see Table 4), 30.6% of these 232 developed cachexia (types 1–3). We were especially interested in the patients with loss of appetite prior to initiation of therapy; 14 (63.6%) of these 22 (4.3% of the 509) patients developed cachexia. There seems to be a trend to develop cancer cachexia more frequently at more advanced tumour stages (Table 1). However, the small numbers precluded a valid statistical analysis.

**Table 4:** Change of appetite and cachexia status (as defined in *Methods*) and *MC4R *103Ile allele carrier status

**Patients with**	**Total** **N^1 ^= 499** **[N = 479]**	**Cachexia** **N = 105 *(21.1)*** **[N = 100]**	**No cachexia** **N = 394 *(78.9)*** **[N = 379]**	**Heterozygotes for the 103Ile- allele** **N = 24 *(4.8)*** **[N = 22]**	**Homozygotes for the Val103-allele** **N = 475 *(95.2)*** **[N = 457]**
**Loss of appetite:**	**232 *(46.5)*** **[223]**	**71 *(67.6)*** **[69]**	**161 *(40.9)*** **[154]**	**9 *(37.5)*** **[8]**	**223 *(46.9)*** **[215]**
General, not further specified	95 *(19)* [91]	37 *(35.2)* [36]	58 *(14.7)* [55]	4 *(16.7)* [3]	91 *(19.1)* [88]
Prior to initiation of therapy	22 *(4.4)* [21]	14 *(13.3)* [14]	8 *(2.1)* [7]	2 *(8.3)* [2]	20 *(4.2)* [19]
During therapy	115 *(23.1)* [111]	20 *(19.1)* [19]	95 *(24.1)* [92]	3 *(12.5)* [3]	112 *(23.6)* [108]
**Occurrence of both food craving and revulsion**	**18 *(3.6)*** **[18]**	**2 *(1.9)*** **[2]**	**16 *(4)*** **[16]**	**1 *(4.2)*** **[1]**	**17 *(3.6)*** **[17]**
**Increase of appetite/no change**	**249 *(49.9)*** **[238]**	**32 *(30.5)*** **[29]**	**217 *(55.1)*** **[209]**	**14 *(58.3)*** **[13]**	**235 *(49.5)*** **[225]**

^1^N = 499 [479]: Of the 509 patients ten without information were excluded, because the patients did not give any information or could not remember their appetite.

Numbers in square brackets denote the number of patients without early leucaemic preliminary stages.

*Numbers in parentheses denote the particular percentage*.

Among 249 patients who described an increased or an unaltered appetite, only 32 (12.8%) developed cancer cachexia. 18 of the total of 509 patients complained about experiencing both phases of craving and revulsion. Finally, 10 of all patients could not give any information pertaining to appetite. Exploratively, we tested for an association between cancer cachexia status (types 1–3 vs. types 4,5) and changes of appetite in two groups (loss of appetite vs. increase of appetite/no change with the former group comprising loss of appetite in general, prior to initiation of therapy or during therapy and occurrence of both food craving and revulsion; see Table 5a; Fisher's exact test, two-sided, p = 9.2 × 10^-6^).

### The role of changes in appetite in Ile103 carriers

Finally, we examined whether there might be an association between changes of appetite defined as above in two groups and the 103Ile allele of the Val103Ile polymorphism. 14 of the 25 heterozygous patients (56%, one of them was cachectic) did not suffer from decreased appetite. One patient could not give any information. The remaining ten patients (40%, one of them was cachectic) stated loss of appetite, three of them explained that the decrease was due to the therapy, one experienced both food craving and revulsion, two patients reported a loss of appetite prior to initiation of therapy, none of them developed cachexia. Exploratively, both without and upon exclusion of the two cases with preliminary leukaemic stages we did not detect a significant association between changes of appetite and the 103Ile allele of the Val103Ile polymorphism (see Table 4; Fisher's exact test, two-sided both p > 0.39).

## Discussion

The Ile103 allele of the Val103Ile polymorphism of the *MC4R *gene has repeatedly been reported in several studies with a large number of cases and controls to be negatively associated with obesity and increased BMI [21-23].

In the light of the postulated role of the MC4R in development of cachexia as determined in different rodent studies, we hypothesized that heterozygotes for the 103Ile allele are more prone to develop cancer cachexia. Surprisingly, our study did not provide any evidence for such an association (p = 0.13). Exploratory post-hoc analyses excluding patients with early leukaemic stages even revealed that there might be a trend towards the opposite effect (p < 0.05); heterozygotes for the 103Ile allele developed cancer cachexia less frequently in comparison to the rest of the study group. If this observation is not simply due to the usual problems of post-hoc subgroup analyses [36], one may speculate that there is a negative correlation between the Val103Ile polymorphism and cancer cachexia in patients with non-hematological/solid malignant neoplasms. This hypothesis has to be tested in additional studies.

If there is indeed a 'protective effect' conferred by the 103Ile allele that prevents patients with cancer from developing cachexia, it would be of interest to determine the underlying mechanisms. It is assumed that the Ile103 allele itself (or another variant in tight linkage disequilibrium) leads to a more active MC4R in functional terms [21]. Since endogenous agonist binding properties and cell surface receptor expression levels are normal for this Val103Ile polymorphism, it had been difficult to link the Ile103 allele with a potential molecular effect. Recently, it was however, observed that the 103Ile MC4R showed a modest (2-fold) but statistically significant decrease in antagonist hAGRP(87–132) potency, which is consistent with a protective effect conferred by this variant [24]. The effect of β-MSH, a potent agonist at the MC4R [25], seemed, on the other hand, to be increased for the 103Ile-allele [24]. Hence, both, the lower antagonist and the increased agonist potencies are compatible with an elevated MC4R function, which could explain the weight reducing effect of the variant. However, these functional findings [23-25], which are compatible with an elevated MC4R function, cannot explain the possible protection against the development of cachexia. There might be differences in binding of these or other endogenous ligands receptor-expression, in signal-transduction and/or physiological conditions [21].

Other explanations include: (i) There might be cachexia inducing factors that are produced by the tumour or resulting from host-tumour-interactions that preferentially react with the Ile103 variant. Deactivation or altered binding properties might ensue. This mechanism might lead to a reduced signalling of the *MC4R *expressing cells and thus result in an inborn resistance to the development of cancer cachexia. (ii) Alternatively, in the presence of cancer certain factors/conditions might alter the receptor, so that binding properties for an antagonist (e.g. AGRP) might improve. (iii) Acute-phase-protein-levels are elevated in patients developing cancer cachexia [37], possibly the interaction between one or a combination of these with the variant MC4R leads to the described effects.

To clarify if the risk of Ile103 carriers for development of cancer cachexia and potentially other forms of cachexia differs from wild type carriers, further studies in patients with solid tumors, and in comparison to non-solid malignant neoplasms are obviously warranted. Due to the carrier frequency in the range of 2–4% this evidently requires large and appropriately characterized patient samples.

Not surprisingly, an exploratory sub-group analysis revealed that changes of appetite (including loss of appetite in general, prior to initiation of therapy or during therapy and occurrence of both food craving and revulsion) might be associated with cancer cachexia (p < 0.01). This description is in line with previous studies [3,35]. Interestingly, many patients could not concretize, some patients could not give any information pertaining to appetite pertaining to appetite; thus it was difficult to separate. We were especially interested in the patients with loss of appetite prior to initiation of therapy; 63.6% of these developed cachexia. However, loss of appetite does not seem to be necessary for developing cachexia, as already described in the past [4]. Finally, we also obtained no evidence for an association of 103Ile carrier status and changes of appetite (p > 0.39).

## Conclusion

In sum, our data do not support the hypothesis of a higher frequency of the 103Ile allele in patients developing cancer cachexia; contrariwise the frequency was descriptively higher in patients not developing cachexia. Thus, the role of the Val103Ile polymorphism of the *MC4R *for the development of cancer cachexia needs further clarification. Nevertheless, our data provide investigators with a basis to develop new hypotheses.

## Competing interests

The author(s) declare that they have no competing interests.

## Authors' contributions

SK, SZ and AH carried out the molecular genetic studies, participated in the sequence alignment, drafted the manuscript and participated in the design of the study. AS performed the statistical analysis. JH and AN conceived of the study, and participated in its design and coordination. All authors read and approved the final manuscript.

## Pre-publication history

The pre-publication history for this paper can be accessed here:


